# P051 Diagnostic Challenge of Takayasu arteritis in a patient with inflammatory bowel disease

**DOI:** 10.1093/rap/rkag113.081

**Published:** 2026-11-03

**Authors:** Muhammad Waleed Tahir, Stephanie Gall, Devishri Shetty, Kiran Putchakayala

**Affiliations:** Mid Cheshire Hospitals NHS Foundation Trust, Crewe, United Kingdom; Mid Cheshire Hospitals NHS Foundation Trust, Crewe, United Kingdom; Mid Cheshire Hospitals NHS Foundation Trust, Crewe, United Kingdom; Mid Cheshire Hospitals NHS Foundation Trust, Crewe, United Kingdom

## Abstract

**Introduction:**

Takayasu arteritis is a rare large-vessel vasculitis that predominantly affects women under the age of 50 years. Early diagnosis can be challenging due to non-specific symptoms, such as malaise and fatigue, in the early systemic phase that often precedes the occlusive phase. We present the case of a 46-year-old woman with a history of mild Crohn’s disease who had recurrent hospital admissions with raised inflammatory markers and symptoms initially attributed to Crohn’s disease. Following thorough reassessment, she was subsequently diagnosed with Takayasu arteritis.

**Case description:**

A 46-year-old woman with a history of mild Crohn’s disease, diagnosed at the age of 34 and never requiring immunosuppression, was referred to rheumatology following recurrent hospital admissions with markedly raised inflammatory markers, constitutional symptoms, and intermittent loose stools. Recent colonoscopy and abdominal imaging showed no evidence of active Crohn’s disease. During her admission she had significantly elevated inflammatory markers (CRP 301 mg/L, ESR 125 mm/h) that slightly improved with presumed Crohn’s flare-up treatment and needed high dose prednisolone for complete resolution.

Further history revealed profound fatigue, nausea, headache, dizziness, night sweats, reduced appetite, and generalised malaise. She described longstanding Raynaud’s phenomenon affecting her hands, photosensitive erythematous rash affecting her left cheek, intermittent chest tightness and breathlessness that improved with steroids and neck pain suggestive of carotidynia. There was no joint pain, digital or oral ulcers, skin thickening, nasal crusting, or other features suggestive of systemic connective tissue disease or ANCA-associated vasculitis.

Examination revealed marked blood pressure difference between both arms (178/96 mmHg on the right and 141/95 mmHg on the left), with an absent left radial pulse. There was mild soft tissue swelling around both ankles without evidence of synovitis and livedo over the knees. No neurological deficits were identified, and cardiovascular and respiratory examinations were unremarkable.

Autoimmune serology including ANA, ENA, ANCA, MPO and PR3 was negative. Hepatitis B, hepatitis C, syphilis and tuberculosis screening were also negative. CT and MRI showed periaortic haziness. PET-CT showed increased FDG uptake in the aorta and left common carotid artery, with subtotal occlusion of left carotid artery, consistent with large vessel vasculitis. She was initially started on a high dose of prednisolone followed by a gradual taper and was subsequently commenced on methotrexate and tocilizumab. Clopidogrel was also started following stroke team discussion, and vascular MDT advised medical management.

**Discussion:**

This case highlights the diagnostic challenge of Takayasu arteritis, especially in its early inflammatory phase. The patient’s longstanding diagnosis of Crohn’s disease created the potential for diagnostic anchoring, with raised inflammatory markers, loose stools and constitutional symptoms repeatedly attributed to inflammatory bowel disease. However, several clinical features were inconsistent with active Crohn’s disease and suggested a systemic inflammatory process. These included Raynaud’s phenomenon, carotidynia, constitutional symptoms, headaches, steroid-responsive chest tightness and recurrent inflammatory state with no evidence of active Crohn’s disease. The most significant clinical findings were the inter-arm blood pressure difference and absent left radial pulse.

In retrospect there were some clues pointing towards large vessel vasculitis. Patient was a paramedic student and had noticed that her colleagues were unable to palpate her left radial pulse a year ago. PET-CT confirmed active inflammation of the aorta and left common carotid artery, supporting the diagnosis of Takayasu arteritis.

This case also highlights a rare but recognised association between inflammatory bowel disease and Takayasu arteritis. Whether the patient’s gastrointestinal symptoms were related to Crohn’s disease or represented an ischaemic manifestation of vasculitis remains unclear.

The patient had subtotal occlusion of the left carotid artery, placing her at significant risk of stroke. This emphasises how extensive vascular involvement may develop before the diagnosis is established and highlights the importance of early recognition and prompt immunosuppressive treatment to reduce complications.

While conventional CT demonstrated periaortic changes, PET-CT confirmed active inflammation in large vessels and the extent of vascular involvement, demonstrating the importance of PET-CT in making a diagnosis. This also helped in starting aggressive immunosuppression and referral for vascular input.

Finally, this case emphasises the importance of a thorough history and physical examination, as well as the need to reevaluate previous diagnoses when clinical findings fail to support them.

**Key learning points:**

1. Diagnostic Anchoring

This case highlights the importance of recognising diagnostic anchoring in patients with an established diagnosis. Patients with existing autoimmune conditions may develop additional inflammatory diseases, and new symptoms should be carefully evaluated rather than automatically attributed to the existing diagnosis.

2. Recognising Takayasu arteritis in early phase

Takayasu arteritis often presents with vague constitutional symptoms during its early inflammatory phase before vascular features become evident in the later occlusive phase. Careful history taking and examination remain the cornerstone of diagnosis. Simple bedside assessments, such as measuring blood pressure in both arms and assessing for pulses, can provide important diagnostic clues.

3. Correlation between investigations and clinical findings

This case also reinforced the importance of correlating clinical findings with objective investigations rather than relying solely on a previous diagnosis. In patients with persistent systemic inflammation despite apparent remission of another inflammatory condition, clinicians should maintain a broad differential diagnosis and consider large-vessel vasculitis where appropriate.

4. Role of PET CT

PET-CT is important in identifying active inflammation and, in this case, revealed active vascular inflammation when conventional CT and MRI findings were subtle. It is important not to exclude large vessel vasculitis based on serology and conventional CT alone.

5. Multidisciplinary Management

Management of Takayasu arteritis requires a multidisciplinary approach. Collaboration between rheumatology, vascular surgery, radiology and stroke services was essential in this case to ensure timely diagnosis and appropriate treatment.

